# Combining Aspirin with Cholecalciferol (Vitamin D_3_) – A Potential New Tool for Controlling Possum Populations

**DOI:** 10.1371/journal.pone.0070683

**Published:** 2013-08-09

**Authors:** David R. Morgan, Jane Arrow, Mark P. Smith

**Affiliations:** 1 Landcare Research, Lincoln, Canterbury, New Zealand; 2 Canterbury District Health Board, Christchurch, Canterbury, New Zealand; University of Sydney, Australia

## Abstract

The introduced Australian brushtail possum is a major vertebrate pest in New Zealand, with impacts on conservation and agriculture being managed largely through poisoning operations. Cholecalciferol (vitamin D_3_) is registered for use in controlling possums and despite its many advantages it is expensive and relatively inhumane. Combination of a high proportion of aspirin with a low proportion of cholecalciferol was effective in killing high proportions of groups of acclimatised, caged possums: this is attributed to both an unexpectedly high toxicity of the type of cholecalciferol used, and a proposed synergistic mechanism between the two compounds. Death was caused by localised damage to heart ventricles by aspirin, and inhibition of tissue repair by both aspirin and cholecalciferol. The observed toxicosis had lower impact on the welfare of possums than either compound administered alone, particularly aspirin alone. Residue analyses of bait remains in the GI tract suggested a low risk of secondary poisoning by either compound. The combination of cholecalciferol and aspirin has the potential to meet key requirements of cost-effectiveness and humaneness in controlling possum populations, but the effect of the combination in non-target species has yet to be tested.

## Introduction

The brushtail possum (*Trichosurus vulpecula*) is a nocturnal, arboreal, largely herbivorous marsupial about the same size as the domestic cat. It was introduced over 150 years ago from Australia to New Zealand to establish a fur trade, and has become a major pest, causing extensive damage to conservation values, agricultural crops and forest plantations, and by spreading bovine tuberculosis [1]. The most commonly used method of control for the last 50 years has been poisoning using sodium fluoroacetate (1080) baits, distributed aerially or from the ground [2]. After many years of public opposition to the use of 1080 baits, particularly by aerial application [3], the practice was formally reassessed by ERMA (Environmental Risk Management Authority, the governmental organisation tasked with assessing the environmental risks associated with using vertebrate pesticides). Although continued use was endorsed with some conditions, a recommendation was made for development of alternative methods that address concerns over the impact of 1080 on environmental and human safety, and its impact on the welfare of both the target and non-target animals [4].

In addition to the frontline pesticide 1080, other compounds have been or are used for possum control in New Zealand. Baits containing cholecalciferol (i.e. vitamin D_3_) have been available for this purpose since its development as a possum poison in the 1990s [5]. At that time, cholecalciferol was sourced from Holland and had an oral LD_50_ in possums of 16.8 mg kg^−1^ [6]. Poisoning occurs through absorption of cholecalciferol from the intestine, conversion to 25–hydroxycholecalciferol in the liver, and subsequent conversion in the kidney to 1,25-dihydroxycholecalciferol, the biologically active form of the vitamin [7]. The latter metabolite works in conjunction with the parathyroid hormone to release calcium from storage in bone and other tissues and to reabsorb calcium in the kidney [8], leading to a maximum concentration of blood calcium in possums at about 4 days [9]. The blood hypercalcaemia then leads to excessive calcification in soft tissues including blood vessels, and death from heart failure in most possums after 4–7 days [9].

Cholecalciferol is highly suitable for possum control due to: (1) its low toxicity to birds (an especially important characteristic in New Zealand where conservation of endemic avian species is often the aim of pest control), (2) a low risk of secondary poisoning of humans, cats and dogs; (3) short persistence in sub-lethally poisoned animals; and, (4) availability to the general public without the need for a licence [10]. However a major limitation is its relatively high cost, which exceeded NZ$3000/kg in 2010. A further disadvantage of cholecalciferol poisoning is that it has significant impact on possums’ welfare before death. Possums typically lose appetite around 24 hours after consuming a lethal dose and eat very little over the next few days, during which they may experience pain due to calcification of soft tissues before dying after 5–6 days [11]. The overall impact of cholecalciferol poisoning in possums was assessed as ‘extreme’ by an expert panel that used available data to classify the relative humaneness of vertebrate pest control tools used in New Zealand [12].

One approach to improving cost-effectiveness is to seek relatively inexpensive compounds that enhance the toxicity of cholecalciferol, thus enabling a reduction in the active concentration used in baits. Such compounds would be particularly useful if they also reduced the welfare impacts of poisoning. The effectiveness of cholecalciferol is likely to be greater when calcium concentration in the blood is high. While addition of calcium carbonate to oral doses of cholecalciferol increased toxicity [6] it subsequently proved unsuitable for use as a bait additive as it is hygroscopic, and the resultant increase in moisture content caused baits to become crumbly and less palatable to possums. Furthermore, it did not accelerate toxicosis or benefit welfare, with time until death remaining at around 7 days.

An alternative approach to achieving higher calcium concentration in the blood is to manipulate the mechanisms that regulate blood calcium. Aspirin is an inexpensive compound (∼ NZ$4/kg) that, once converted from salicylic acid to salicylate, causes metabolic acidosis of the blood in mammals [13], a condition in which reduced serum pH promotes the release of calcium bound to albumin into physiologically active ionised calcium [14], [15]. It has been suggested that cholecalciferol could be combined with anticoagulant pesticides such as diphacinone or coumatetralyl in order to reduce the concentration of these persistent compounds and the consequent hazard to wildlife presented by bioaccumulation [16]. However, aspirin is unlikely to present such a hazard as it is rapidly metabolised and excreted in mammals: for example, in humans seven oral doses of 1 g at six hourly intervals were cleared in less than 48 h [17], and oral doses of 44 mg kg^−1^ were more than 90% eliminated from goats and cattle in 24 h, and in 12 h from rabbits [18]. While no previous investigations of aspirin with cholecalciferol as a pesticide were located in the scientific literature, one lapsed patent (Patent number 0392934/EP-B1) was found that proposed the combination for more effective rodent control.

We therefore investigated key characteristics of the combination of cholecalciferol and aspirin to assess its potential for further development. Toxicity and efficacy were assessed using groups of captive possums given oral doses of cholecalciferol, and aspirin, separately and in combination. Feeding trials were conducted to establish if high concentrations of (inexpensive) aspirin with low concentrations of (expensive) cholecalciferol would result in high mortality and improved humaneness. The secondary-poisoning risk to animals that may scavenge possum carcasses was assessed by tracing the fate of dyed bait in the intestinal tract and assaying residual levels of cholecalciferol and aspirin in dyed digesta. The relative humaneness of different combinations of aspirin and cholecalciferol was determined from observation of the progression of toxicosis through eight defined states of responsiveness. The mode of toxic action was investigated by serial measurement of a range of clinical parameters following dosing, and by gross pathological examination and histology of selected tissues in poisoned possums.

## Methods

### Ethics Statement

All studies were conducted in compliance with the New Zealand Animal Welfare Act (1999) under the approvals (numbers 08/09/02 and 11/03/02) and monitoring of the Landcare Research Animal Ethics Committee.

### Materials

Cholecalciferol was obtained from a commercial source (Zhejiang Garden Biochemical High-Tech Stock Co., China) and was assayed by HPLC to determine purity, prior to use [19]. The level of purity was identified by identifying all peaks in the HPLC graph, and calculating the proportional area under peaks for compounds other than cholecalciferol. Cholecalciferol accounted for 87.8% (95% CI = ±5%) of the mass of substance, and the concentrations of prepared dose solutions were therefore corrected accordingly. The main impurity was 2,5–hydroxycholecalciferol, the first metabolite produced following absorption of cholecalciferol. Aspirin was used in the form of acetylsalicylic acid powder (Sigma-Aldrich NNZ, Auckland, product no. A5376) and was also assayed by HPLC for purity [20]. Doses selected were expected to be high enough to demonstrate an effect on serum calcium while avoiding significant toxicity. Initially, aspirin was used at 500 mg kg^−1^, which is approximately half the LD_50_ for rats (i.e. 920 mg kg^−1^) [21].

Possums were trapped on private land at Glenhope Station in the Lewis Pass region of New Zealand’s South Island with the land-owner’s permission. Cage traps, activated by a treddle mechanism, were baited with a lured dough mixture and checked each morning after they were set. Captured possums were transferred to hessian sacks and transported to a purpose-designed animal facility where they were acclimatised indoors for at least 4 weeks in individual cages (530×400×1000 mm) containing separate nest boxes (230×200×350 mm). They were maintained on a diet of fruit, vegetables and supplementary feed pellets, and reticulated water was continually available. Ambient temperature was maintained within the range 18−22°C, and ventilation provided for complete refreshment of air every 4 min. Lighting in the animal facility followed the normal outdoor seasonal cycle being controlled by a light sensor outside the building. Possums that survived the doses administered or ingested in trials were anaesthetised with isoflurane delivered at 5% in oxygen (flow rate of 3 L min^−1^) and then humanely euthanased with an intra-cardiac overdose of 150 mg kg^−1^ of sodium pentobarbitone.

### Oral Toxicity Trials

The acute toxicity to possums of cholecalciferol, aspirin, and cholecalciferol combined with aspirin was assessed over a range of doses using groups of six possums at each dose (Tables 1, 2, and 3, and Tables S1, S2, and S3). Cholecalciferol toxicity was determined, following gavage delivery of test substances, from seven groups of possums dosed at 0, 1, 2, 4, 8, 16, or 64 mg kg^−1^. Aspirin toxicity was determined from four groups of possums dosed at 250, 500, 1000, or 2000 mg kg^−1^. Cholecalciferol toxicity was then reassessed at 0, 1, 2, 4, or 8 mg kg^−1^ in combination with a constant dose of aspirin of 1000 mg kg^−1^. A further assessment was made using 4 or 16.8 mg kg^−1^ cholecalciferol combined with 500 mg kg^−1^ aspirin. Possums were randomly assigned to treatment groups which comprised equal numbers of males and females. Oral dosing was accomplished by anaesthetising possums with isoflurane and gently passing a 7 mm-diameter plastic tube containing the appropriate dose solution through the mouth into the stomach. The required volume of dose solutions was then administered by a 50-ml syringe fitted to the tube. As cholecalciferol is insoluble in water, peanut oil was used as the dosing vehicle in a dose volume of 3 ml kg^−1^.

**Table 1:** Oral dosing with cholecalciferol alone.

Treatment group	Cholecalciferol dose(mg kg^−1^)	Mortality			Mean time to death (days)	Mean change in body weight of possums killed (%)
		Male	Female	Total		
1	0	0/3	0/3	0/6	–	–
2	1	0/3	0/3	0/6	–	–
3	2	0/3	0/3	0/6	–	–
4	4	1/3	2/3	3/6	8.7	−18.4
5	8	2/3	1/3	3/6	6.0	−13.7
6	16	3/3	2/3	5/6	5.8	−16.3
7	64	3/3	3/3	6/6	5.0	−11.6

Mortality of males and female possums after dosing with cholecalciferol, mean time until death, and mean change in bodyweight of possums that died. Treatment group 1 received the dose solution containing no cholecalciferol. Group 6 received the approximate LD_50_ dose [6].

**Table 2:** Oral dosing with aspirin alone.

Treatment group	Aspirin dose(mg kg^−1^)	Mortality			Mean time to death (days)	Mean change in body weight of possums killed (%)
		Male	Female	Total		
1	0	0/3	0/3	0/6	–	–
2	250	0/3	1/3	1/6	6	−19.4
3	500	0/3	0/3	0/6	–	–
4	1000	0/3	1/3	1/6	0.7	−6.0
5	2000	0/3	2/3	2/6	0.7	−10.9

Mortality of males and female possums after dosing with aspirin, mean time until death, and mean bodyweight change. Treatment group 1 received the dose solution containing no aspirin.

**Table 3:** Oral dosing with combinations of cholecalciferol and aspirin.

Treatment group	Cholecalciferol dose(mg kg^−1^)	Aspirin dose (mg kg^−1^)	Mortality			Mean time to death (h)	Mean change in body weight of possums killed (%)
			Male	Female	Total		
1	0	0	0/3	0/3	0/6	–	–
2	1	1000	3/3	1/3	4/6	12.0	−12.7
3	2	1000	2/3	2/3	4/6	23.5	−4.1
4	4	1000	2/3	2/3	4/6	19.5	−9.1
5	8	1000	3/3	3/3	6/6	12.0	−7.5

Mortality of males and female possums after dosing with cholecalciferol combined with aspirin at a constant dose, mean time until death, and mean change in bodyweight.

The effect of dosing on serum calcium concentration was monitored in four groups of six possums dosed with (i) 16.8 mg kg^−1^ cholecalciferol (as the nominal LD_50_ value for possums [6]), (ii) 16.8 mg kg^−1^ cholecalciferol combined with 500 mg/kg aspirin (iii) 500 mg kg^−1^ aspirin and (iv) the peanut oil dose vehicle as a control. Since dosing with cholecalciferol alone resulted in maximum serum calcium after about 4 days [9], doses were designed to ensure sufficient possums survived to enable comparison of serum calcium between treatment groups over time. Blood samples were collected 8 d before dosing, and at 4, 8, 16, and 24 h, and 2, 3, 4, 6, 8 and 10 d after dosing. Blood was collected from the tail vein of isoflurane-anaesthetised possums using a 25-gauge needle. At each sample point, 2 ml of blood was taken from three possums, with the three remaining possums in each treatment group blood-sampled at the next point, thus alternating sampled possums so that blood-sampling did not exceed ethical limits i.e. no more than 7.5% of total blood volume within 24 h [22]. Serum was separated by centrifuging, and stored at −20°C until assay of calcium by standard spectrophotometric methods (Canterbury Health Laboratories, Christchurch).

We postulated that addition of aspirin would, compared with cholecalciferol alone, increase mortality, further increase serum calcium, and reduce mean time until death and mean change in bodyweight between dosing and death (or the 21-day end-point for survivors). Comparisons were made using one-sided t-tests. To estimate LD_50_ values we used the generalised linear models procedure in the R statistical computing environment (version 2.1.11) with a binary indicator 0/1 for dead or alive, respectively, to estimate mortality as the dependent variable, and the independent variable was the dose of cholecalciferol or aspirin or cholecalciferol with 500 mg kg^−1^ of aspirin. Regression curves of mortality against dose were fitted for both cholecalciferol and aspirin as the independent variables where the dose of the other substance was zero, and LD_50_ estimates taken from the fitted curves.

### Bait Consumption/Efficacy Trials

Trials were conducted to establish (i) the maximum concentration of aspirin that naïve possums would accept in baits, and (ii) the minimum concentration of cholecalciferol needed in paste bait to give high (i.e. >90%) mortality when combined with the selected concentration of aspirin (i.e. 20% wt:wt). (Note: all further % concentration values are on the basis of wt:wt).

#### Maximising aspirin

Possums were randomly assigned to six treatment groups which each comprised five males and five females. Aspirin at 0, 10 and 20% was offered in non-toxic paste bait, either ‘PestOff’ apple-based paste (Animal Control Products Ltd, Whanganui) and a peanut-based paste (Pest Control Research Ltd, Christchurch) (i.e. six treatments altogether). Possums were presented, for 16 h overnight, with 100 g of a test treatment and 100 g cereal-based RS5 cereal pellet baits (a highly palatable bait formulation produced by Animal Control Products Ltd, Whanganui) as a ‘reference’ material (which was novel to all possums) for estimation of the relative palatability of test treatments. Weights consumed were calculated after weighing remaining bait and correcting for the average moisture loss recorded for three reference samples of bait placed adjacent to possum cages. ‘Palatability’ (to each animal) was calculated as the mass of test treatment consumed expressed as a percentage of the combined consumption of test and reference treatments. The effect of aspirin on bait palatability was determined by ANOVA and linear regression.

#### Minimising cholecalciferol

‘Response-guided’ trials were conducted to determine the minimum concentration of cholecalciferol needed to achieve >90% mortality. The first of five groups of possums (*n* = 10, equal sex ratio) was presented with peanut-based paste nominally containing 0.8% wt:wt cholecalciferol (i.e. the concentration registered for use in New Zealand) as a control treatment. Paste was presented for 16 h overnight and in addition to normal food rations. The second group received paste nominally containing 0.025% cholecalciferol: this concentration of cholecalciferol, which is only 3% of the registered concentration for use in possum baits, was selected as a starting point since the oral dosing trials showed that 1 mg kg^−1^ (i.e. 6% of the LD_50_) resulted in 4/6 deaths when combined with 1000 mg kg^−1^ of aspirin. Three further groups were then presented with paste nominally containing 0.05, 0.1, and 0.2% cholecalciferol. Weights consumed were calculated after weighing remaining bait and correcting for moisture loss as described above, and doses of cholecalciferol and aspirin ingested by assaying three randomly collected samples of each paste formulation for both substances.

### Assessing Risk of Secondary Poisoning to Scavengers

During the ‘response-guided’ trials, limited sampling was undertaken to estimate the fate and concentration of cholecalciferol and aspirin in the possum gut. All paste treatments also contained 0.1% rhodamine dye (‘Been there’, FIL Industries, Mount Maunganui, New Zealand) as a bait marker shown previously to have no effect on bait palatability [23]. Possums dosed with cholecalciferol alone (n = 6) or the combination with aspirin (n = 5) were autopsied at death to gain an indication of how widely the paste was distributed in the intestinal tract. Staining of gut contents was obvious only in the latter group: samples from the stomach and upper small intestine were taken from two possums in that group and assayed for concentration of cholecalciferol and aspirin.

### Assessing Welfare Impact

The impact of dosing on possums’ welfare was assessed for four groups of possums (each n = 6). The doses given, by oral intubation as described above, were: group 1, 5 mg kg^−1^ cholecalciferol; group 2, 1.3 g kg^−1^ aspirin; group 3, 5 mg kg^−1^ cholecalciferol and 1.3 g kg^−1^ aspirin; and group 4, 10 mg kg^−1^ cholecalciferol and 1.3 g kg^−1^ aspirin.

At specified intervals (see below), each possum was monitored by direct observation of posture and responsiveness to stimuli using the following classifications [11].

0 No effect – normal responses and alertness

1 Slight effect – slightly unbalanced with some uncoordination in movement

2 Moderate effect – moderately unbalanced, severe uncoordination, can stand or sit upright but reluctant to do so

3 Down but responds – lying on back, unable to stand but easily aroused (e.g. by sound)

4 Little response – lying on back or side, little response to stimuli (i.e. touch and sound)

5 Light anaesthesia – lying on side and unable to lie on back, or move back to nest box, responds only to painful stimuli (i.e. pinching between the digits)

6 Anaesthetised – no response to painful stimuli

7 Death.

Unusual behaviours observed during monitoring, such as vomiting or abnormal breathing, were also recorded observed.

The sampling intervals used differed between groups. Time until death was expected to be around 5–7 days for possums dosed with cholecalciferol alone. These possums were therefore assessed twice daily until they reached Stage 1 on the above scale, after which they were monitored 2-hourly until they reached stage 2 when observation intervals were reduced to 15 min. Possums in the other two groups were expected to die more quickly and were initially monitored at 2-hour intervals. Once possums reached Stage 2, sampling frequency was increased to intervals of 15 min. Observations were not made during the hours 19∶30 to 08∶00 resulting in incomplete observation of the latter stages of toxicosis in two possums dosed with cholecalciferol.

### Mode of Action

Necropsies were performed by a veterinary pathologist (Gribbles Veterinary Pathology, Christchurch) on five possums that died after oral dosing with 10 mg kg^−1^ cholecalciferol and 1.3 g kg^−1^ aspirin. Gross pathological examination of all organs (including the brain) identified heart and lung tissues as the only tissues consistently showing visible irregularities in all specimens, and these were further examined histologically.

A clinical investigation was also undertaken to help elucidate the mode of action when cholecalciferol is combined with aspirin. Three groups of possums (each n = 12) were orally dosed, as described above, with either cholecalciferol alone (5 mg kg^−1^), aspirin alone (1.3 g kg^−1^), or combination of these doses. Blood samples (2 ml) were collected, as described above, alternately from subgroups of 6 possums at 24 h before dosing, and at 5, 10 and 24 h after dosing such that blood collection from individuals did not exceed ethical limits [22]. Samples were also collected from animals observed to be comatose. All samples were analysed by Canterbury Health Labs (Christchurch) using standard clinical assays. In addition to monitoring serum calcium and salicylate, blood clotting time (using ‘activated partial thromboplastin time’ (APTT)) and fibrinogen were monitored to identify anticoagulant activity of aspirin, while troponin I was monitored as this protein is a specific marker of heart tissue damage [24], [25] occurring in all vertebrates [26]. Heart rate was also measured at each sample point using a Microlife BP A100 Automatic Blood Pressure monitor.

### Cost-effectiveness

Current prices of cholecalciferol and aspirin were obtained to assess the change in cost using differing concentrations of the two substances. The bulk price of cholecalciferol manufactured in China was NZ$674/kg (as of 21/11/12): corrected for the measured impurity of 11%, this is equivalent to NZ$754/kg of pure compound. Aspirin powder, also sourced from China, costs NZ$3.78/kg (mean of two quotations received November 2012), and purity is generally >99%. Using these prices, the predicted cost reduction relative to the present lowest concentration of cholecalciferol registered for possum and rodent control (0.4%) was modelled for combinations of cholecalciferol at 0.4–0.025% and aspirin at 0–25%. This analysis was also conducted to estimate potential reduction in the price of bait products using a value of NZ$2.16/kg for pellet baits (Animal Control Products, Wanganui, as of 1/10/12). The concentrations of the two compounds predicted to be most cost-effective by the bait consumption trials were used to assess potential reduction in cost.

## Results

### Oral Dosing Trials (Gavage Treatment)

#### Toxicity

The lowest dose of cholecalciferol alone that resulted in deaths was 4 mg kg^−1^, while 5 of 6 possums were killed by 16 mg kg^−1^ (Table 1 and Table S1). Death resulted in approximately equal proportions of males (9 of 18) and females (8 of 18). The estimated LD_50_ was 6.4 mg kg^−1^ (95% CI: 4.3–9.6 mg kg^−1^) with males at 5.7 mg kg^−1^ (3.0–11.1) not significantly different from females at 7.1 mg kg^−1^ (3.7–13.5). The mean time until death for possums from all dose groups combined was 6.8 days (SE = 0.9). The mean change in bodyweight at death was −14.6% (SE = 1.7%) while control animals gained an average 4.6% (SE = 2.6%) in bodyweight between dosing and the end of the trial. There was a weak inverse correlation between dose and time to death (r = 0.17, p = 0.1, df. = 16) and reduction in body weight of possums that died (r = 0.19, p = 0.08, df. = 16), suggesting higher doses caused quicker deaths with less weight loss.

Aspirin alone was lethal to four out of 12 female possums, at a dose as low as 250 mg kg^−1^ (Table 2 and Table S2). Three died around 16 h after dosing while one possum died six days after dosing. Possums that died lost 11.8% bodyweight on average. The estimated LD_50_ was 1359 mg kg^−1^, and although this estimate is statistically imprecise (95% CI: 801–4661 mg kg^−1^), it suggested a fixed dose of aspirin of 1000 mg kg^−1^ as a starting point for assessing combination with cholecalciferol. No male possums died at any dose of aspirin.

Deaths of both males (10 of 12) and females (8 of 12) occurred in all treatment groups receiving combinations of cholecalciferol and aspirin (Table 3 and Table S3). The estimated LD_50_ of cholecalciferol combined with a fixed dose of 1000 mg kg^−1^ aspirin was 0.65 mg kg^−1^. As this was, unexpectedly, below the range of doses tested, further testing at cholecalciferol doses lower than 1 mg kg^−1^ would be needed for determination of the LD_50_ within an estimated confidence interval. Nevertheless, the combination of aspirin with cholecalciferol proved very effective especially when compared with Dutch-sourced cholecalciferol (LD_50_ = 16.8 mg kg^−1^ [6]), and it was more toxic than the Chinese-sourced cholecalciferol alone (6.4 mg kg^−1^) used in this study.

Possums died, on average, 16.4 h after receiving the aspirin-supplemented doses (range for individual possums = 2–45 h). This was significantly less than the 7 days found for cholecalciferol alone (t = 10.0, d.f. = 33, p<0.001), and with significantly less reduction in bodyweight before death (i.e. 8.2% v. 14.5%) (t = 3.3, d.f. = 33, p = 0.001).

#### Blood calcium

**Figure 1:**
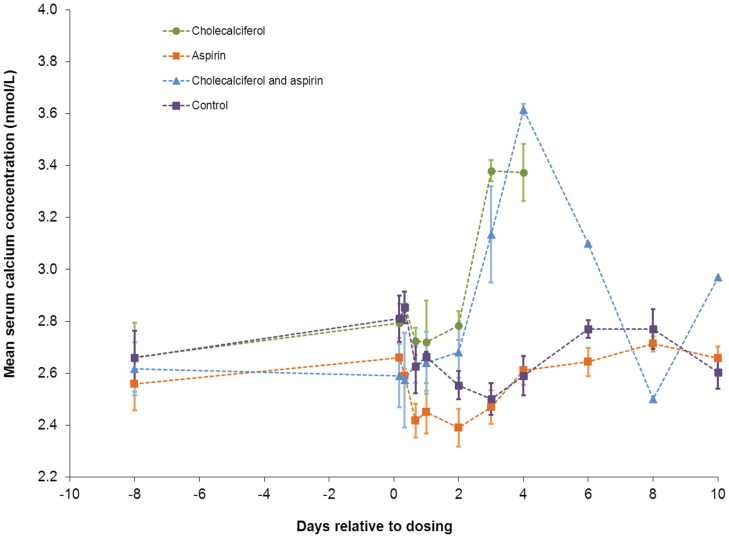
Effect of oral dosing with cholecalciferol and aspirin, alone and combined, on possums’ serum calcium. Mean (±SE) serum calcium concentration (nmol/l) before and after groups of possums (each n = 6) were orally dosed with 16.8 mg kg^-1^ cholecalciferol and 500 mg kg^-1^ aspirin alone and in combination, and dosing vehicle alone (control group). The three data points the cholecalciferol/aspirin group from days 6–10 represent single values. Dotted lines were fitted by eye to indicate trends.

Mortalities occurred in the cholecalciferol group (6/6) and the combination group (5/6) between 1–8 days after dosing, reducing availability for blood sampling over time. The data obtained show that serum calcium increased following dosing with cholecalciferol (16.8 mg kg^−1^) alone and combined with aspirin (500 mg kg^−1^), reaching a peak concentration at around 4 days (Fig. 1 and Table S6). However, combination with aspirin did not lead to earlier or higher peak concentration, and when aspirin was administered alone (500 mg kg^−1^), serum calcium concentration decreased for two days after dosing relative to control values (t = 2.6, d.f. = 8, p = 0.02).

### Bait Consumption Trials

#### Maximising aspirin

**Figure 2:**
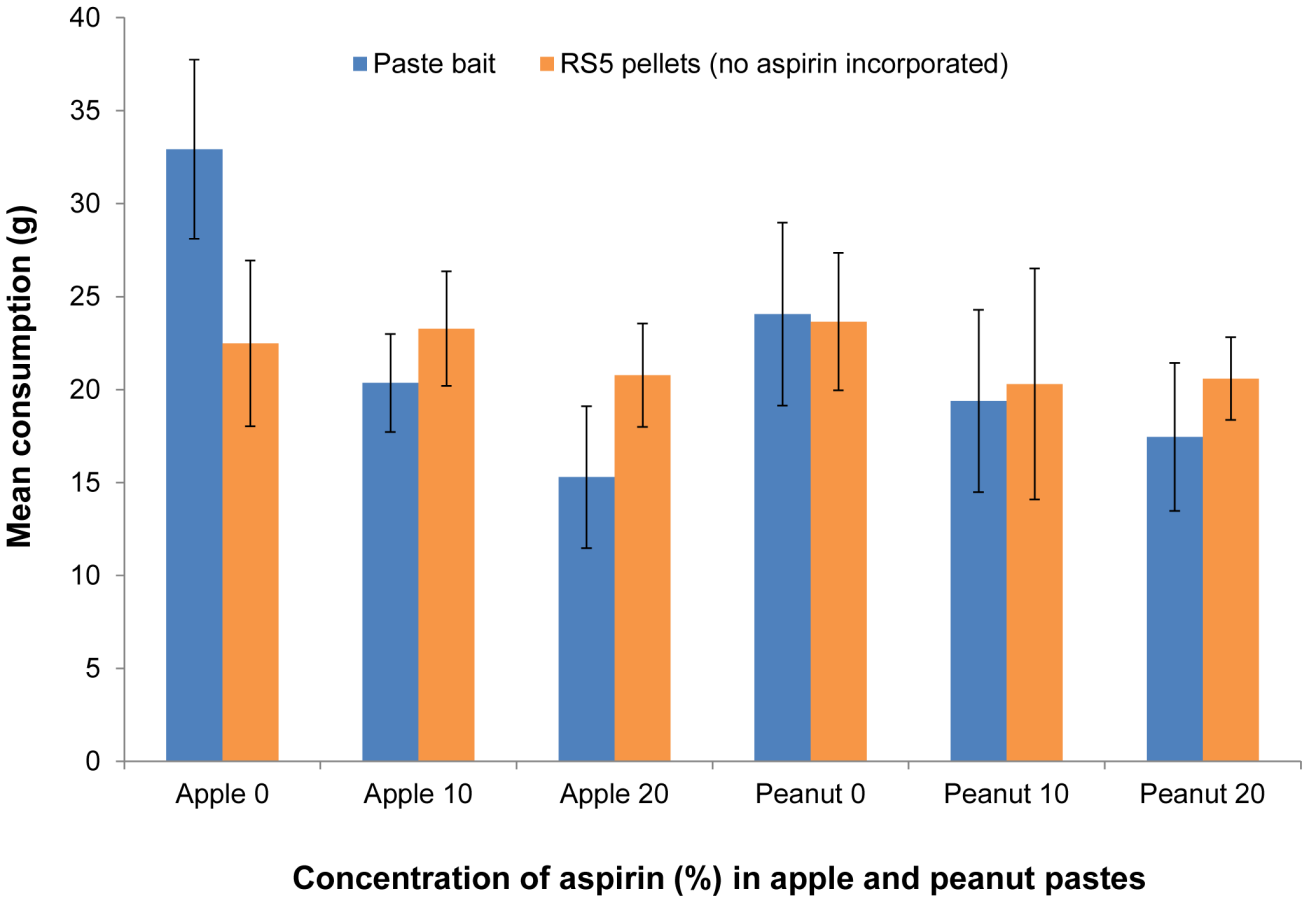
Effect of aspirin on possums’ consumption of paste bait. Mean weight (±SE) eaten (g) by possums of two types of paste bait, each incorporating aspirin at 0, 10 or 20%; and their consumption of RS5 cereal pellets used as a ‘reference’ bait. Each pair of bars indicate the two treatments offered to six different groups of possums (each n = 10).

Overall, mean consumption of apple or peanut paste bait alone (i.e. disregarding the effect of aspirin) was not significantly different (Paired t test_(2 tail) = _0.38, d.f. = 29, p = 0.71), nor was the consumption of cereal pellets (Paired t test_(2 tail) = _0.21, d.f. = 29, p = 0.83) (Fig. 2 and Table S7). When consumption was expressed as palatability (i.e. as a percentage of the combined consumption of paste and pellets), aspirin reduced palatability (F_1,57_ = 4.80, p = 0.03) with an average decrease in palatability of 0.72% for every 1% increase in aspirin concentration. However, even at 20% aspirin, mean consumption of pastes was 15 g or more, but because the minimum amounts eaten by individual possums were 0 g for apple paste and 5.1 g for peanut paste, peanut paste was selected as the vehicle for further bait consumption trials using 20% aspirin.

#### Minimising cholecalciferol

Assays of the actual (as opposed to ‘nominal’) concentrations of cholecalciferol in paste (Table 4) enabled accurate calculation of doses ingested. All possums died after eating 0.86% cholecalciferol (without aspirin) (Table 4 and Table S4), with the mean dose ingested (113.6 mg kg^−1^) being considerably greater than the LD_50_ (6.4 mg kg^−1^) estimated in the oral gavage dosing trials. Lower cholecalciferol concentrations (0.026–0.23%) combined with 20% aspirin produced 60–90% mortality, but no correlation was found between mortality and concentration of toxicants used. This is because the dose ingested is dependent not only on the concentrations of toxicants, but also the palatability of each formulation and consequent quantity eaten. The mean time until death was reduced from 5.4 days (SE = 0.8 days) for group 1 to 23.6 h (SE = 6.5 h) for group 5, with a reduction in the body-weight lost from 10.4% to 4.0%. However, this excludes one outlier in group 5 that died after 6 days, and lost 16.8% weight.

**Table 4:** Possums response to paste bait with combinations of cholecalciferol and aspirin.

Treatment group,in order of testing	Chole. concn (% wt:wt)	Aspirin concn(% wt:wt)	Mean wt (g)bait eaten	Mean dosechole (mg kg^−1^)	Mean dose aspirin(mg kg^−1^)	Mortality			Mean time until death	Mean % change inbody wt
						Male	Female	Total		
1	0.86	0	37.2	113.6	0	5/5	5/5	10/10	5.4 days	−10.4
2	0.026	20	33.3	2.6	2220	4/5	4/5	8/10	Not monitored	−6.7
3	0.054	20	27.5	6.3	2060	2/5	4/5	6/10	Not monitored	−4.3
4	0.11	20	34.0	14.8	2690	4/5	5/5	9/10	Not monitored	−6.7
5	0.23	20	25.7	21.1	1830	4/5	3/5	7/10	23.6 hours*	−4.0

Mean values for consumption of peanut paste bait, toxicant doses, mortality, time until death, and change in bodyweight after dosing with Chinese cholecalciferol of 88.4% purity combined with 20% wt:wt aspirin.

The asterisk indicates the exclusion of one outlier in group 5 that died after 6 days, and lost 16.8% weight.

**Figure 3:**
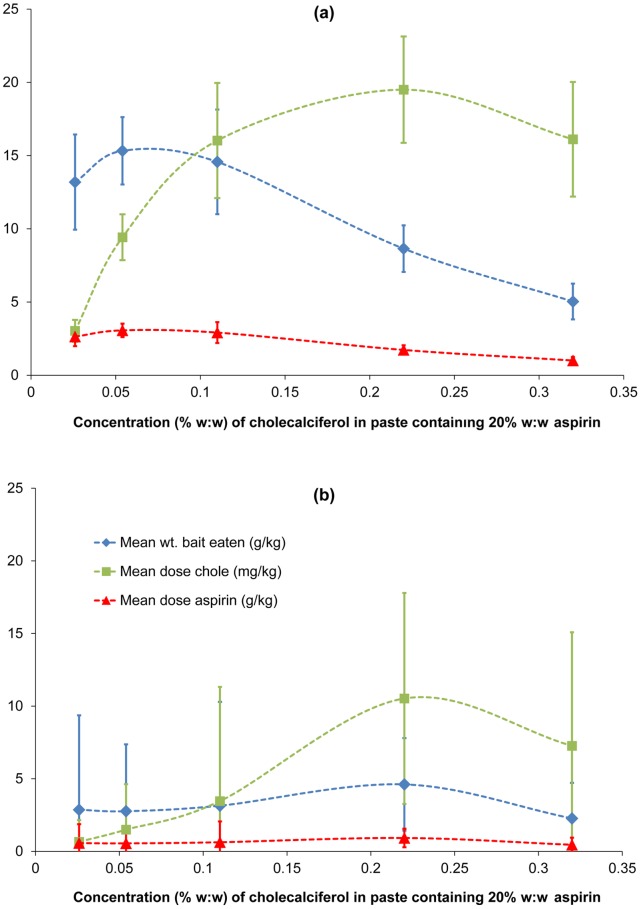
Effect of cholecalciferol on possums’ consumption of paste with 20% aspirin. Five groups of possums (each n = 10) were each presented with paste bait containing aspirin at 20% and cholecalciferol at 0.026–0.86%. Mean (±SE) bait consumption (g) and doses of cholecalciferol (mg/kg) and aspirin (g/kg) ingested are shown separately for possums that died (a) survived (b). Dotted lines were fitted by eye to indicate trends. The vertical axes apply to the three parameters given in the legend.

For possums that were killed (Fig. 3a and Table S8), amounts of paste and aspirin ingested were similar for cholecalciferol treatments up to 0.11%, but above this concentration, mean consumption of paste and consequent mean aspirin dose declined, suggesting that bait palatability was reduced by the increasing cholecalciferol concentration. This resulted in an increase in the mean dose of cholecalciferol ingested up to a concentration of around 0.2%, but a decline in the associated aspirin dose ingested. At 0.1% cholecalciferol and 20% aspirin, mean doses of 15 mg kg^−1^ cholecalciferol and 2.7 g kg^−1^ aspirin were ingested, and minimum ingested doses were 6.5 mg kg^−1^ and 1.2 g/kg respectively.

Survivors in all dose groups sampled only a small quantity of paste bait (2–3 g) before rejecting it (Fig. 3b and Table S8). The cholecalciferol doses of 3.5–13.3 mg kg^−1^ ingested by survivors in the 0.11% and 0.23% cholecalciferol treatment groups exceeded the doses (1.8–3.0 mg kg^−1^) ingested by seven of the eight possums killed in the 0.026% cholecalciferol group, but the associated aspirin doses ingested were less (630–1200 mg kg^−1^ vs.1550–2600 mg kg^−1^). This suggests that, for high mortality, minimum doses of 13 mg kg^−1^ of cholecalciferol and 1500 mg kg^−1^ of aspirin are needed, and this would be delivered by 15 g of peanut paste bait containing 0.1% cholecalciferol and 20% aspirin.

### Assessing Risk of Secondary Poisoning

Inspection of gut contents after death revealed a clear difference between the two groups of possums sampled. Five of six Group 1 possums (see Table 4 for treatment details) showed some residual red-staining of both the paste-like contents of the caecum and the more solid pellets in the colon. The sixth possum showed no rhodamine traces, most probably because death occurred one day later than other possums, therefore allowing more time for bait residue to be eliminated. By contrast, four Group 5 possums (see Table 4 for treatment details) that had consumed paste and died in 19 h or less all contained rhodamine-dyed stomach contents that were liquid in texture. Aspirin is known to be a stomach irritant and it is possible that possums had drunk more water than usual in response to this irritation: indeed, two Group 5 possums were seen drinking during scheduled observation periods. Assays for residues in Group 5 possums showed that cholecalciferol was rapidly absorbed since concentrations were much lower in the contents of the small intestine compared with the stomach of the two possums from which samples were assayed (Table 5). Aspirin concentrations were similar in the contents of the stomach and small intestine suggesting that absorption occurs in both, as in humans [27].

**Table 5:** Toxicant residues in the GI tract.

Possum no.	Sex	Total dose ingested		Digesta assayed	Digesta concentration	
		Cholecalciferol (mg)	Aspirin (g)		Cholecalciferol(mg g^−1^)	Salicylate (mg g^−1^)
4	Female	51.9	10.4	Stomach contents	0.371	0.61
5	Male	39.8	7.8	Stomach contents	0.506	1.16
4	Female	51.9	10.4	Small intestine	0.004	1.06
5	Male	39.8	7.8	Small intestine	0.009	1.08

Doses of toxicants ingested and their concentration in the stomach and small intestine for two possums presented with paste containing 0.23% cholecalciferol and 20% aspirin.

### Assessing Welfare Impact

When aspirin was combined with cholecalciferol, the mean time to death was greatly reduced from 4.8 days (group 1) to 15–19 hours (groups 3 and 4) (Table 6 and Table S5) and weight loss was correspondingly reduced, confirming the earlier result from bait consumption trials (Table 4). Deaths occurred in 8 of 14 males and 8 of 10 females. Efficacy was highest in group 4 where the combined doses were closest to the required minimum doses estimated in the bait consumption trials (i.e. 13 mg kg^−1^ cholecalciferol and 1.5 g kg^−1^). Efficacy of aspirin alone at 1.3 g kg^−1^ was higher than recorded in earlier dosing trials (Table 2), although mean time to death was longer: since the same type of aspirin was used throughout all trials, it is likely that difference reflects the small sample sizes used in these trials.

**Table 6:** Mortality of possums after dosing with cholecalciferol combined with aspirin at a constant dose, mean time until death, and mean change in bodyweight.

Treatment group	Cholecalciferol dose(mg kg^−1^)	Aspirin dose (g kg^−1^)	Mortality			Mean time to death (hours)	Mean change in body weight (%)
			Male	Female	Total		
1	5	0	1/4	1/2	2/6	114.7	−13.5
2	0	1.3	2/3	3/3	5/6	29.6	−7.6
3	5	1.3	2/4	1/2	3/6	15.2	−4.4
4	10	1.3	3/3	3/3	6/6	19.1	−5.5

**Figure 4:**
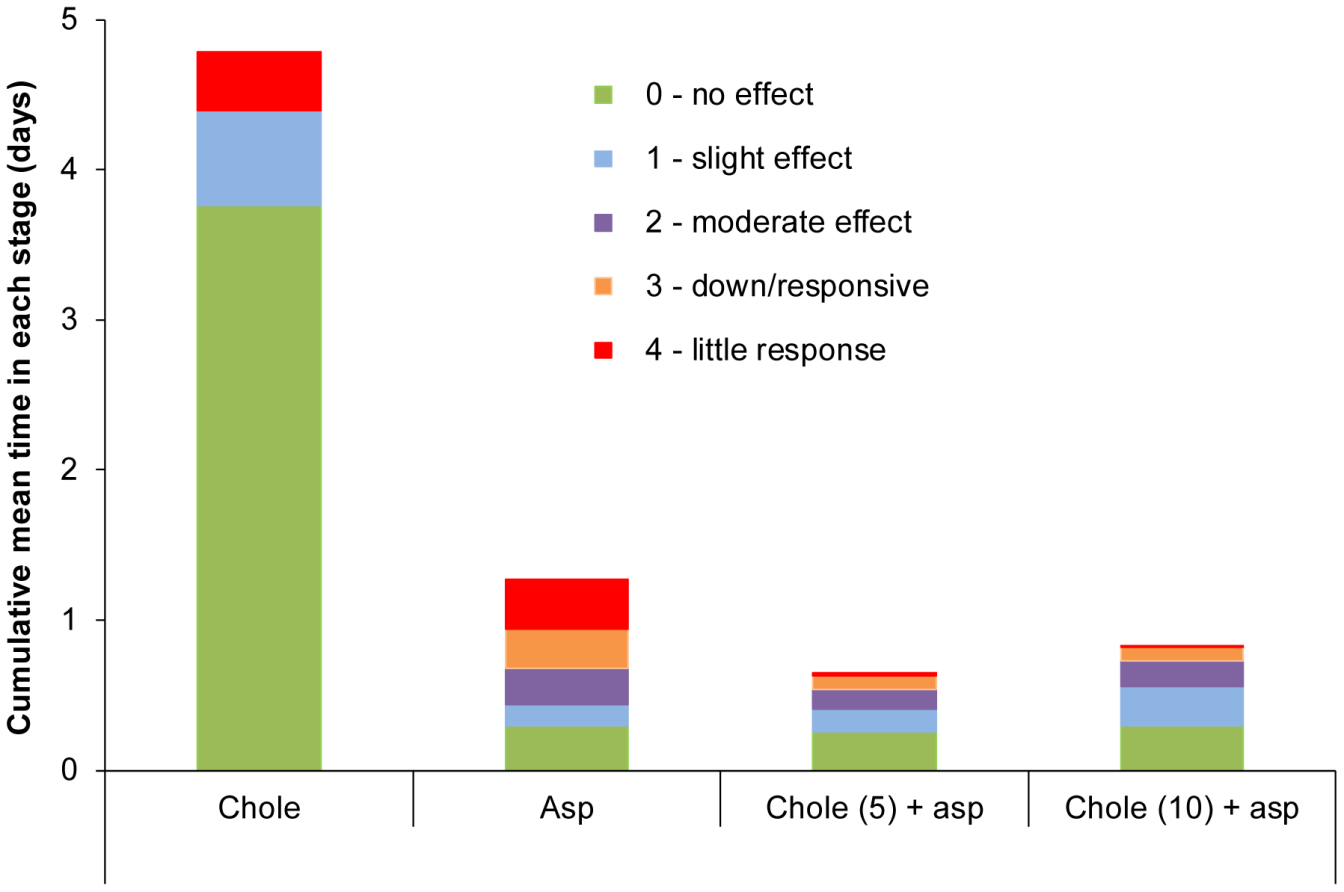
Relative humaneness of cholecalciferol and aspirin, alone and in combination, in poisoning possums. The cumulative mean time spent in five of the eight defined stages (0–7) for possums that died following dosing with (i) 5 mg kg^−1^ cholecalciferol (abbreviated as ‘Chole’), ii) 1.3 g kg^−1^ aspirin (‘Asp’), iii) 5 mg kg^−1^ cholecalciferol and 1.3 g kg^−1^ aspirin, and iv) 10 mg kg^−1^ cholecalciferol and 1.3 g kg^−1^ aspirin. Six possums were observed in each treatment group. The green sections indicate ‘no visible effect’ (stage 0) while other colours combined indicate the progression of toxicosis (i.e. stages 1–4) during which possums were conscious and visibly affected by the dosing. Possums progressed directly from stage 4 to death (stage 7), which is indicated by the top of each bar.

In summary, for possums that died the mean length of toxicosis experienced while conscious (i.e. during stages 1–4 inclusive) was 24.5 h for possums dosed with cholecalciferol alone, 23.3 h for aspirin, 9.3 h for aspirin with 5 mg kg^−1^ cholecalciferol, and 12.9 h for aspirin with 10 mg kg^−1^ cholecalciferol (Fig. 4 and Table S9). No possums were observed in stages 5 or 6, although two possums dosed with cholecalciferol alone were not observed in the final stages of toxicosis (which occurred during the non-observed period between 19∶30 and 08∶00 h) resulting in possible underestimation of the welfare impact of cholecalciferol. However, all other possums in groups 2–4 were observed to progress directly from stage 4 to death: these possums were observed to die quite suddenly while fully alert without progressing through a prolonged phase of being moribund. All animals that were dosed with aspirin, alone or in combination, were observed breathing more heavily than usual during the conscious phase of toxicosis, and seven of these possums showed increased salivation (two in group 2 and five in group 4). No vomiting, retching, or unusual vocalisation was noted in any treatment group, but one possum dosed with the combination developed very dilated pupils.

These results therefore indicate a synergy of cholecalciferol with aspirin resulting in a quicker death with a reduced period of welfare compromise than by either compound alone. Aspirin alone may cause a prolonged period of welfare compromise, but in combination with cholecalciferol, this is reduced.

### Mode of Action

#### Pathology and histology

All possums that died after oral dosing with 10 mg kg^−1^ cholecalciferol and 1.3 g kg^−1^ aspirin were in good condition with abundant body fat, and irregularities were observed only in the heart and lungs of all individuals. Gross examination found reddening and congestion of the myocardium, most markedly in the sub-epicardium of the left and right ventricles. Histological examination showed all animals had intense congestion in the lungs. This condition is usually accompanied by the accumulation of fluid in the bronchial passages but, since there was no evidence of this, it is unclear if the congestion occurred before or after death. In the absence of any other observed irregular pathology, these observations suggested that possums died as a result of an acute cardiac arrhythmia leading to cardiac failure.

#### Clinical measurement

**Figure 5:**
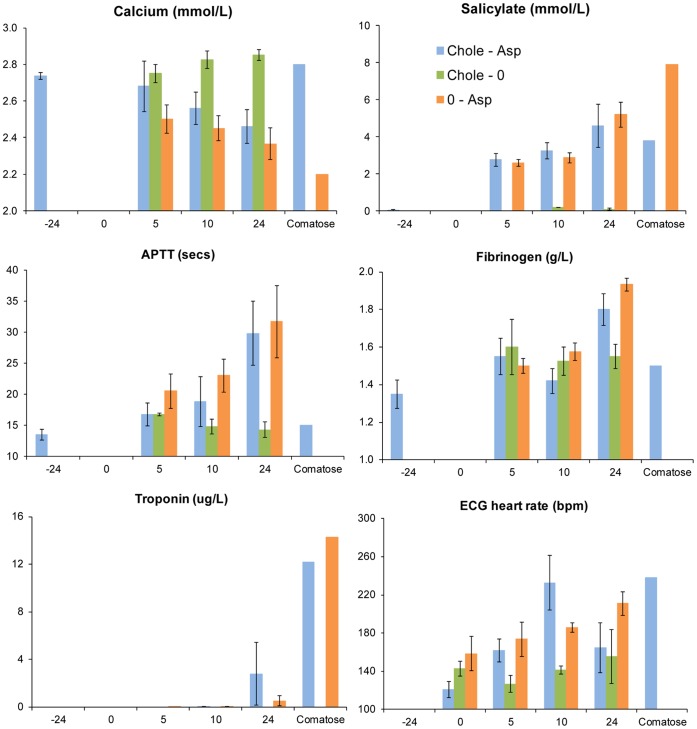
Blood parameters monitored before and after oral dosing possums with cholecalciferol and aspirin, alone and in combination. Serum concentrations of calcium, salicylate, fibrinogen, and troponin I; and activated partial thromboplastin time and ECG heart rate. Units in captions apply to the vertical axes. Mean values and standard error bars are shown for measurements taken before and after dosing with cholecalciferol (5 mg/kg), aspirin (1.3 g/kg), or these doses combined as indicated by the abbreviations shown in the ‘Salicylate’ part of the figure. Note the horizontal axis (hours relative to dosing) is non-linear and also indicates measurements taken from two comatose possums (dosed with the combination and aspirin alone) shortly before death at 14.1 and 25 h.

All blood parameters showed a response to dosing with aspirin (alone or in combination with cholecalciferol) while dosing with cholecalciferol alone did not affect the parameters monitored (Fig. 5 and Table S10). The mean serum calcium concentration remained stable relative to pre-dose values in possums dosed with cholecalciferol during the 24-h monitoring period, consistent with earlier findings (Fig. 1). Possums receiving aspirin (alone or with cholecalciferol) showed declining serum calcium, although one comatose possum receiving the combination showed normal serum calcium. Salicylate levels increased over 24 hours in possums receiving aspirin, and salicylate was present in comatose animals. Impaired blood clotting following aspirin-dosing was clearly indicated by the APTT tests by 5 h following dosing, while fibrinogen concentration increased between 10 and 24 h after dosing with aspirin. Myocardial damage by aspirin, consistent with the pathological evidence, was indicated by the presence of troponin at the 24-h measurement, while heart rate became elevated in aspirin-dosed possums by the 10 h measurement, reaching up to twice the normal rate, even in a comatose possum.

### Cost-effectiveness

**Figure 6:**
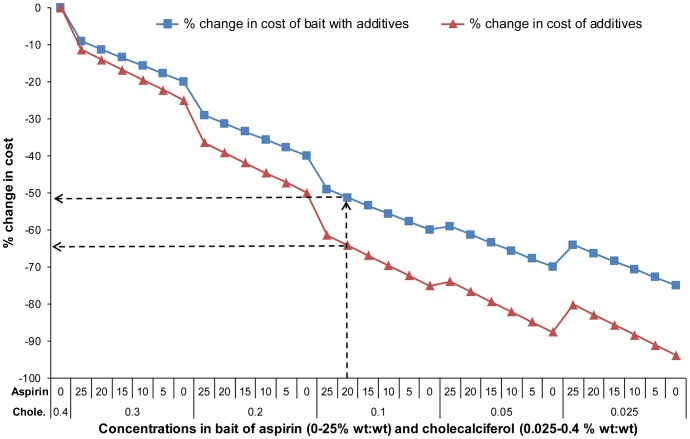
Potential cost-savings given by combinations of cholecalciferol and aspirin. Estimated potential reduction in price of toxic pellet baits and toxicants alone relative to current cost by combining cholecalciferol with aspirin at varying concentrations. The arrows indicate potential savings at the optimal concentrations identified in this study.

Modelling the cost of combining varying proportions of the two components showed there is considerable scope for reducing the price of cholecalciferol bait products by combination with aspirin (Fig. 6 and Table S11).

The combination of 0.1% cholecalciferol with 20% aspirin that we found in bait consumption trials to be optimal in terms of palatability and effectiveness offers a cost-saving of about 65% compared with the presently-registered lowest concentration of cholecalciferol of 0.4% used in possum baits [28]. When combined in cereal pellet bait the saving was estimated to be about 50%.

## Discussion

Continued progress in eradicating bovine tuberculosis [29] and restoring conservation values [30] in New Zealand is presently underpinned by a strategic approach to the control of invasive vertebrate pests, a major component of which is the use of 1080 poison to control possum populations [31]. In response to the ERMA recommendation for alternatives to 1080 [4], we undertook an initial evaluation of the combination of cholecalciferol and aspirin as an alternative toxicant. A wide range of studies is needed to fully evaluate any proposed, new alternative, the key criteria being: cost-effectiveness, humaneness, stability and suitability for bait delivery, risks to non-target species and environmental persistence. Our initial investigations of the critical criteria suggest that the combination has considerable potential for use in possum control. The combination was effective in killing high proportions of groups of acclimatised, caged possums: this is attributed to, both, an unexpectedly high toxicity of the type of cholecalciferol used, and a proposed synergistic mechanism between cholecalciferol and aspirin. The observed toxicosis had lower impact on the welfare of possums than either compound administered alone, particularly aspirin. Use of the optimum concentrations of the two compounds that we identified should help overcome the cost-based resistance to use of cholecalciferol baits to date in possum control.

### Toxicity

The estimated LD_50_ for Chinese-manufactured cholecalciferol of 6.4 mg kg^−1^(95% CI: 4.3–9.6 mg kg^−1^) was considerably lower than the estimate of 16.8 mg kg^−1^ (95% CI: 11.6–21.9 mg kg^−1^) reported by Jolly et al. [9]. The purity of Chinese cholecalciferol used in this and other studies was within a range of 81–89%, while earlier development of cholecalciferol as a possum toxin [9] used Dutch-sourced cholecalciferol that was found by our lab to have purity typically exceeding 95%. Limited lab analyses indicated that Chinese cholecalciferol contained a larger proportion of the first metabolite, 25–hydroxycholecalciferol, than Dutch cholecalciferol. This is likely to result in more rapid accumulation of the second metabolite 1,25–dihydroxycholecalciferol, the more biologically active form of the vitamin, which binds to vitamin D receptors at a rate 1000 greater than cholecalciferol [7] (but is far too costly to use as a pesticide).

The peak serum calcium concentrations we recorded were similar to the 3.6–3.8 nmol l^−1^ reported earlier [9] following cholecalciferol doses of 10 mg kg^−1^. This was as expected since serum calcium concentration is known to be tightly regulated by interactions between calcium, 1,25–dihydroxycholecalciferol and the parathyroid hormone controlling fluxes of calcium in bone, kidneys and the intestine [32]. However, we did not record earlier peaking of serum calcium suggesting that the greater toxicity of Chinese cholecalciferol is attributable to a cause other than, or in addition to, the presence of a greater amount of its first metabolite. Two other impurities indicated by the manufacturer’s certificate of purity, tachysterol (comprising 14.3% of the substance) and lumisterol (3.2%), are biologically inactive. In animals, these are produced to protect against cholecalciferol-induced hypercalcaemia when exposure of the skin to UV light, which converts the plant-derived 7-dehydrocholesterol to cholecalciferol, becomes excessive [33]. More comprehensive assay by, for example GCMS-MS, is required to determine the reason for the greater toxicity of the Chinese cholecalciferol.

### Non-target Secondary Poisoning Risk

Risk of secondary poisoning is conventionally assessed as the product of the ‘hazard’ presented, which is the toxicity of contaminated tissues in possum carcasses, their persistence in this state and the likely ‘exposure’ of non-target animals to this hazard through feeding on carcass tissues. Due to the more rapid mode of action of the combined toxicants compared with cholecalciferol alone, carcasses had visible remains of paste bait in the stomach, and assays showed that cholecalciferol was in higher concentration in the stomach contents than in digesta lower in the gut, while appreciable aspirin residues were found throughout the gut. Consequently, scavenging non-target species, such as dogs, hawks, and pigs are potentially likely to be exposed to toxic residues if they feed on stomach contents.

The potential hazard presented by the individual compounds can be estimated based on the highest concentrations recorded from the limited sampling conducted here. The LD_50_ of cholecalciferol in dogs is 88 mg kg^−1^ [34] and the LD_50_ of aspirin is 2–4 g kg^−1^ [21], so a dog weighing 20 kg would have to consume over 3 kg of stomach contents to be at risk of lethal cholecalciferol poisoning, and at least 40 kg to be at risk of aspirin poisoning. However, risk-assessment in a range of non-target species must be based on the consequence of presenting cholecalciferol and aspirin together, for which no data presently exist.

Further assessment of non-target risk should also consider the residual concentrations of the metabolites of cholecalciferol since 25-hydroxy-cholecalciferol and its derivative, the biologically active 1,25-dihydroxycholecalciferol, may persist in liver and kidney tissues of sub-lethally poisoned (with 0.8% cholecalciferol in bait) possums for several weeks [35]. However, the hazard is likely to be less after dosing with the combination because (i) cholecalciferol would be used at a lower concentration and (ii) the more rapid action of cholecalciferol in combination with aspirin will permit less time for accumulation of metabolites. Furthermore ingestion of the metabolites is likely to result in their partial degradation in the GI tract [35].

### Welfare Impact

As noted by others, it is important that new methods for controlling vertebrate pests should be acceptably humane [36]–[38]. In New Zealand, this is predicated not only on a growing social awareness for more humane pest control, but also on the requirements of the Animal Welfare Act which requires that research to develop new poisons must ensure that animals are killed in such a manner that the animal ‘does not suffer unreasonable or unnecessary pain or distress’. Although no direct measure of pain and distress is possible, our data showed that the animals’ welfare was compromised less by the combination than by its components alone, both in terms of time to death, loss of body mass, appetite, and time experiencing toxicosis. While aspirin caused heavier breathing during toxicosis, any associated pain and suffering may have been ameliorated due to the analgesic property of salicylate, which remained in high concentration in the circulating blood throughout the period until death. Relative to other poisons used for controlling possums in New Zealand, the combination of aspirin and cholecalciferol appears to offer an improvement in humaneness over the use of cholecalciferol alone, and may also be superior to 1080 which, on average, causes 9.5 h of possible pain, weakness and disorientation in possums [39].

### Synergy and Mechanism

A synergy between cholecalciferol and aspirin (at 20%) was indicated by: further reduction of the LD_50_ of cholecalciferol to possums to an estimated 0.65 mg kg^−1^, increased efficacy relative to the same doses of the two compounds alone, a greatly reduced mean time until death, and a reduction in the overall impact on possums’ welfare during toxicosis compared with treatment by the two compounds alone. Although only females were killed by dosing with aspirin alone, there was no gender difference in response to dosing with the combination with mortality in 26 of 34 males (76%) and 24 of 30 females (80%) in the oral dosing and bait consumption trials combined.

While we initially proposed that aspirin would elevate serum calcium, thereby enhancing the activity and lethal effect of metabolised cholecalciferol, an alternative explanation is required since aspirin dosing actually caused a decline in serum calcium. The separate roles in relation to human cardiovascular health of both cholecalciferol [40], [41] and aspirin [42], [43] point towards calcium as a common mediator of the clinical and pathological effects we observed in possums, namely the detection of troponin and impaired coagulation, damage to ventricular heart tissue, and elevated heart rate.

Calcium is involved in regulating both heart muscle contraction [44], [45] and blood coagulation [46], [47]. Regular contraction of heart muscle is dependent on calcium binding and unbinding with the protein troponin on the surface of actin and myosin filaments [45], [48]. The observed aspirin-induced reduction in serum calcium, also found in isolated heart tissue of rats subjected to high concentration of salicylate [49], [50], is likely to have caused a disturbance in the contractility of heart muscle fibres [51]. Disturbances and damage to both the ventricle and atrial tissues of the human heart are reported as a result of salicylate toxicity [52]. Heart muscle tissue is especially vulnerable to low calcium levels because, in addition to the sodium fluxes that regulate contraction of skeletal muscle, rhythmic contraction of the cardiac muscle filaments is dependent also on cyclical fluxes of calcium [44]. Furthermore, salicylate has been shown to inhibit cardiac mitochondrial respiration in isolated heart tissue of rats [53], [54], and such disturbance to cellular energy supply is likely to further impair normal heart function and cause cellular damage, as indicated by troponin being shed from the damaged muscle fibres and released into the serum [55]. The observed elevation of heart and respiration rate is likely to have occurred, under the control of the baroreceptor system, as a response to reduced blood pressure and oxygenation of the blood [56]. Accumulation of fluid in the lungs, as observed in aspirin-treated possums, can occur following salicylate-induced changes in the permeability of blood vessels [57], but the lack of fluid in the bronchial passages suggested that this condition may have developed after death rather than being a contributory cause of death.

Following the initial damage to heart tissue by aspirin, both cholecalciferol and aspirin are likely to inhibit tissue repair through different anticoagulant properties. Blood platelets, which are normally produced as the first stage of tissue repair, must first be activated, then made adhesive before aggregating into clumps through the addition of fibrin. Aspirin is well-known to reduce platelet activation by inhibiting (i) the cyclooxygenase enzyme that controls the formation of the surface attractant molecule thromboxane [52] and (ii) the recognition of tissue factors that signal damage [58]. Impaired coagulation was indicated in possums by the elevated APTT values following aspirin-dosing, and by the accumulation of ‘unused’ fibrinogen that is normally converted to fibrin to form the cross-links between platelets. Further exacerbating this condition, cholecalciferol also directly inhibits one of the tissue factors that is required to activate blood platelets [46], [59] and promotes the breakdown of fibrin [60], [61] especially when serum calcium levels are low [62]. Although serum calcium levels were increased by cholecalciferol when administered alone, this elevation occurred after 3 days, as found previously [9], while possums’ serum calcium remained unchanged during the period until death (approximately 24 hours) when coadministered with aspirin. Thus while cholecalciferol used alone as a possum toxicant induces hypercalcaemia and occlusion of tissues with calcium deposits over a period of around 5 days, in combination with aspirin it serves to inhibit the repair of heart tissue damaged by a hypocalcaemic condition induced by aspirin after around 10–20 hours. Cholecalciferol is known to synergise with another anticoagulant compound, coumatetralyl [63], but this involved a different mechanism in which a plasma coagulation factor was inhibited, resulting in widespread internal haemorrhage, which we did not observe.

We therefore tentatively suggest that the lethal mode of action of the combination of aspirin and cholecalciferol was initiated by aspirin-induced damage to the ventricular muscle tissue and disturbance to heart contractility, with the condition being exacerbated by the inhibition of tissue repair due to the anticoagulant action of both aspirin and cholecalciferol. The consequence is likely to have been inadequate oxygenation of the blood caused by impaired supply from the right ventricle and/or inadequate supply of blood to the tissues caused by impaired supply from the left ventricle, and consequent hypoxaemia leading to heart-failure.

### Conclusions

These investigations suggest that the combination of cholecalciferol and aspirin may better meet some of the key criteria required of vertebrate pesticides than cholecalciferol used alone. While the focus of our work is control of possum populations, cholecalciferol is also used internationally in controlling rodents. There may therefore be potential to improve the efficiency and humaneness of rodent control using the combination as cholecalciferol is also a relatively expensive and inhumane means of controlling rodents [37].

With regard to possum control in New Zealand, the combination would appear to be a suitable candidate to fulfil the ERMA recommendation for the development of alternatives to 1080. Investigations are now needed to both consolidate and advance these initial promising findings. More statistically precise LD_50_ estimates are needed as a fundamental parameter underpinning the further development of the combination. Further data are required comparing the effectiveness of the combination relative to the components alone as the present data are from small sample sizes. The bitter taste of aspirin may result in a small proportion of possums ingesting a sub-lethal quantity of bait, so it would be useful to overcome this limitation through the use of flavourings or an encapsulated form of aspirin. A major question that we have not yet explored is the risk towards non-target species both in terms of their likely exposure (which is dependent on animals’ opportunity, ability and tendency to eat bait) and the hazard this presents in terms of animals’ physiological responses. Bait formulations will also have to be developed with a shelf-life of at least 12 months to avoid wastage, and a field-life of at least several days to facilitate the development of efficient baiting strategies based on minimal deployment of baits. While research to date has mainly used paste bait for its convenience in preparing test formulations, and such a product would be useful for ground-based control of possums, development of solid baits for aerial delivery of the combination should also be a priority.

## Supporting Information

Table S1 **Data for** **Table 1** **.**
(XLSX)

Table S2 **Data for** **Table 2** **.**
(XLSX)

Table S3 **Data for** **Table 3** **.**
(XLSX)

Table S4 **Data for** **Table 4** **.**
(XLSX)

Table S5 **Data for** **Table 6** **.**
(XLSX)

Table S6 **Data for** **Figure 1** **.**
(XLSX)

Table S7 **Data for** **Figure 2** **.**
(XLSX)

Table S8 **Data for** **Figure 3** **.**
(XLSX)

Table S9 **Data for** **Figure 4** **.**
(XLSX)

Table S10 **Data for** **Figure 5** **.**
(XLSX)

Table S11 **Data for** **Figure 6** **.**
(XLSX)
